# Interplay of membrane crosslinking and curvature induction by annexins

**DOI:** 10.1038/s41598-022-26633-w

**Published:** 2022-12-29

**Authors:** Anna Mularski, Stine Lauritzen Sønder, Anne Sofie Busk Heitmann, Mayank Prakash Pandey, Himanshu Khandelia, Jesper Nylandsted, Adam Cohen Simonsen

**Affiliations:** 1 Department of Physics, Chemistry and Pharmacy, University of Southern Denmark, Campusvej 55, 5230 Odense M, Denmark; 2 Membrane Integrity, Danish Cancer Society Research Center, Strandboulevarden 49, 2100 Copenhagen, Denmark

**Keywords:** Membrane biophysics, Atomic force microscopy, Confocal microscopy, Computational biophysics, Membrane curvature

## Abstract

Efficient plasma membrane repair (PMR) is required to repair damage sustained in the cellular life cycle. The annexin family of proteins, involved in PMR, are activated by Ca^2+^ influx from extracellular media at the site of injury. Mechanistic studies of the annexins have been overwhelmingly performed using a single annexin, despite the recruitment of multiple annexins to membrane damage sites in living cells. Hence, we investigate the effect of the presence of the crosslinking annexins, annexin A1, A2 and A6 (ANXA1, ANXA2 and ANXA6) on the membrane curvature induction of annexin A4 (ANXA4) in model membrane systems. Our data support a mechanistic model of PMR where ANXA4 induced membrane curvature and ANXA6 crosslinking promotes wound closure. The model now can be expanded to include ANXA1 and ANXA2 as specialist free edge membrane crosslinkers that act in concert with ANXA4 induced curvature and ANXA6 crosslinking.

## Introduction

The plasma membranes of eukaryotic cells are routinely damaged as cells migrate through the extracellular matrix^1^ or are subject to force, such as those in the muscular, vascular, gastrointestinal or epidermal tissues^2–6^. Eukaryotic cells, therefore, require an efficient mechanism for plasma membrane repair (PMR). While passive phospholipid reorganization can reseal small membrane wounds (< 0.2 μm), larger wounds require active PMR processes due to membrane tension at wound sites caused by the cortical cytoskeleton^7,8^. PMR is triggered when Ca^2+^ influx from the extracellular media enters the cytoplasm at the site of injury. PMR comprises a complex set of processes involving cytoskeletal remodeling, membrane shedding, resupply, and fusion, and many different lipid and protein species^9^. One family of proteins, the annexins, are heavily implicated in PMR. The family has 12 members in humans (ANXA1-11, ANXA13) and is currently of significant therapeutic interest due to their overexpression in various invasive cancers^1^.

The annexins bind reversibly to anionic phospholipids in a Ca^2+^ dependent manner^10^. The N-terminal region is variable in terms of length and sequence^11^, providing the differential binding modes and functions in the protein family^12^. Among all members of the annexin family, the annexin core domain is largely conserved. Localised at the C-terminal region of the protein, the shape of the annexin core domain is a convex disc with membrane attachment sites on the convex side^13^. Thus, membrane association of annexins induces changes in membrane shape and spontaneous membrane curvature.

Observation of membrane patches with free edges after exposure to different annexins has allowed for the characterization of the different resulting curvature morphologies. Similar morphologies were found to coincide with similar amino acid sequences^14^. An example of this can be demonstrated by ANXA4 and ANXA5. They are structurally similar (with 320 and 321 amino acids respectively, where 310 amino acids correspond to the annexin core domain)^4^, and both were found to induce strong curvature in membrane patches with free edges, resulting in a complete, multidirectional roll up of the membrane patch beginning from several points at the membrane patch edge^14^. The proteins have many other similarities too: in the MCF7 breast cancer cell line, ANXA4 and ANXA5 were found to have similar mRNA expression levels, Ca^2+^ sensitivity and recruitment to membrane damage sites^15^; both bind to membranes as trimers, functioning to restrict phospholipids and other protein mobility on the membrane during repair^16^; as trimers, they self-assemble on membranes in 2D crystal arrays^17^; and, both proteins are central to differing mechanistic models of PMR. The capacity of ANXA5 to form 2D arrays is central to the model of PMR proposed by Bouter et al*.*^18^. In the model, membrane bound ANXA5 arrays provide structural support while other repair processes close the lesion. The capacity of ANXA4 trimers to induce membrane curvature is central to the model of PMR proposed by Boye et al*.*^6^. In the model, ANXA4 trimers induce membrane curvature at a lesion’s membrane free edges, resulting in the formation of a ‘neck’. Another member of the annexin family, ANXA6, acts in concert with ANXA4 to promote wound closure at the ‘neck’. ANXA6 is unique in the annexin family, containing two annexin core domains, connected by a flexible linker^19^. This means that the protein can be oriented such that it can bind different membrane areas simultaneously. The capacity of ANXA6 to bind adjacent areas of a membrane at low calcium concentration (~ 60–150 μM) and two separate membranes at high calcium concentrations (~ 2 mM) has been demonstrated in vitro^20^. It has also been reported that ANXA6 repair caps are formed at cellular injury sites^21,22^ suggesting that the role of ANXA6 in PMR is not only based on membrane binding but also on self-interaction in vivo*,* where the capacity for ANXA6 to self-interact has been established in vitro^23^.

In an attempt to reconcile the Boye and Bouter models, the potential cooperativity of ANXA4 and ANXA5 was recently studied^15^. A (1:1) mixture of ANXA4 and ANXA5 was studied alongside ANXA4 and ANXA5 alone (equivalent n). The mixture induced rolling of membrane patches with an intermediate time constant relative to the pure annexins. When exposed to supported lipid bilayers, the (1:1) mixture resulted in a random arrangement of trimers on the membrane surface, in contrast to the crystal arrays of trimers formed by the pure annexins. These findings support the Boye et al. model, as curvature induction was a functional property of the annexin mixture, while the formation of the ANXA5 lattice was not. The implication of this result is that annexins must be considered in combination when proposing mechanistic models, rather than as singular actors. In vivo studies have also suggested that studying annexins in combination may be important. Firstly, it was shown that in cells unable to express ANXA5, PMR was not prevented, just less efficient, due to the activity of other annexins and various other species involved in repair^18^. Secondly, it has been determined that when the membranes of breast cancer cells are injured, the cell surface level of many of the annexins (ANXA1-7, ANXA9 and ANXA11) is increased^24^. Taken together, these studies open up the possibility that the annexins act in concert. To better understand the role of the annexins in PMR in living cells, mechanistic studies of the annexins in combination must be performed.

Like ANXA6, ANXA1 and ANXA2 have been identified as membrane crosslinkers^25^. They have similar sequences, with 346 and 339 amino acids respectively, where 310 amino acids represent the annexin core domain^11^. Upon membrane binding of the annexin core domain, the N terminal regions of both proteins are available as secondary membrane binding sites that promote membrane aggregation^25^. In PMR, both ANXA1 and ANXA2 have been shown to be important not only for membrane crosslinking as monomers, but also as recruiters of additional machineries to complete repair^26^, specifically, by binding as hetero-dimers consisting of two annexins connected via the N-terminus by S100A11 and S100A10 respectively^27^.

The annexins have been found to have differential recruitment to sites of plasma membrane damage which has been correlated to Ca^2+^ sensitivity^28^. However, there is no consensus on the relative recruitment of the annexins to wound sites in studies of different species of living cells. In the laser injury experiments of Boye et al*.*^6^ with MCF7 breast cancer cells, ANXA6-GFP was recruited to the wound site a few seconds before ANXA4-RFP in accordance with their calcium sensitivies^29^. Potez et al*.* found that ANXA6, requiring lower initial [Ca^2+^] for binding to the plasma membrane, responds faster to an injury than ANXA1 in human embryonic kidney, astrocytoma and smooth muscle cells^30^. Several studies of the differential recruitment of ANXA1 and ANXA2 to damage sites in various human and mouse cells found that ANXA1 was recruited before ANXA2^26,27,31^ though this was not found to be the case in zebra fish^32^ or in HEK cells, where ANXA2 was found to be recruited before all other annexins studied, including ANXA1, ANXA6 and ANXA4 which were found to be recruited in agreement with previously cited data, ANXA6 > ANXA4 > ANXA1^33^. These observations suggest species specific differences in the wound site recruitment of different annexins.

In experiments with membrane patches with free edges, ANXA1, ANXA2, and ANXA6 induced folding of membrane patches so that over time, most of the original patch area is converted into folded membrane structures^14^. However, in the case of ANXA1 and ANXA2, the onset of this morphological change was accompanied by blebbing of membrane patch edges, with the folding morphology appearing after the blebbing. For ANXA6, folding is localized at the free edges of the membrane or at visible defect sites in the original membrane. These observations point to alternate mechanisms that result in these different morphologies. These mechanistic studies of annexins have necessarily been performed using a single annexin to determine the function and binding mechanism of specific annexins, but they raise questions about the potential cooperativity or interference of the different annexin species during PMR. To come closer to understanding the concerted mechanism of PMR in living cells, these proteins must be considered in combination. Here, we take a step towards this goal by studying the potential cooperativity of a curvature inducing annexin, ANXA4, with the crosslinking annexins, ANXA1, ANXA2 and ANXA6 in model membrane systems.

## Results and discussion

### Membrane patch rolling induced by mixtures of ANXA4 and the crosslinking annexins

**Figure 1:**
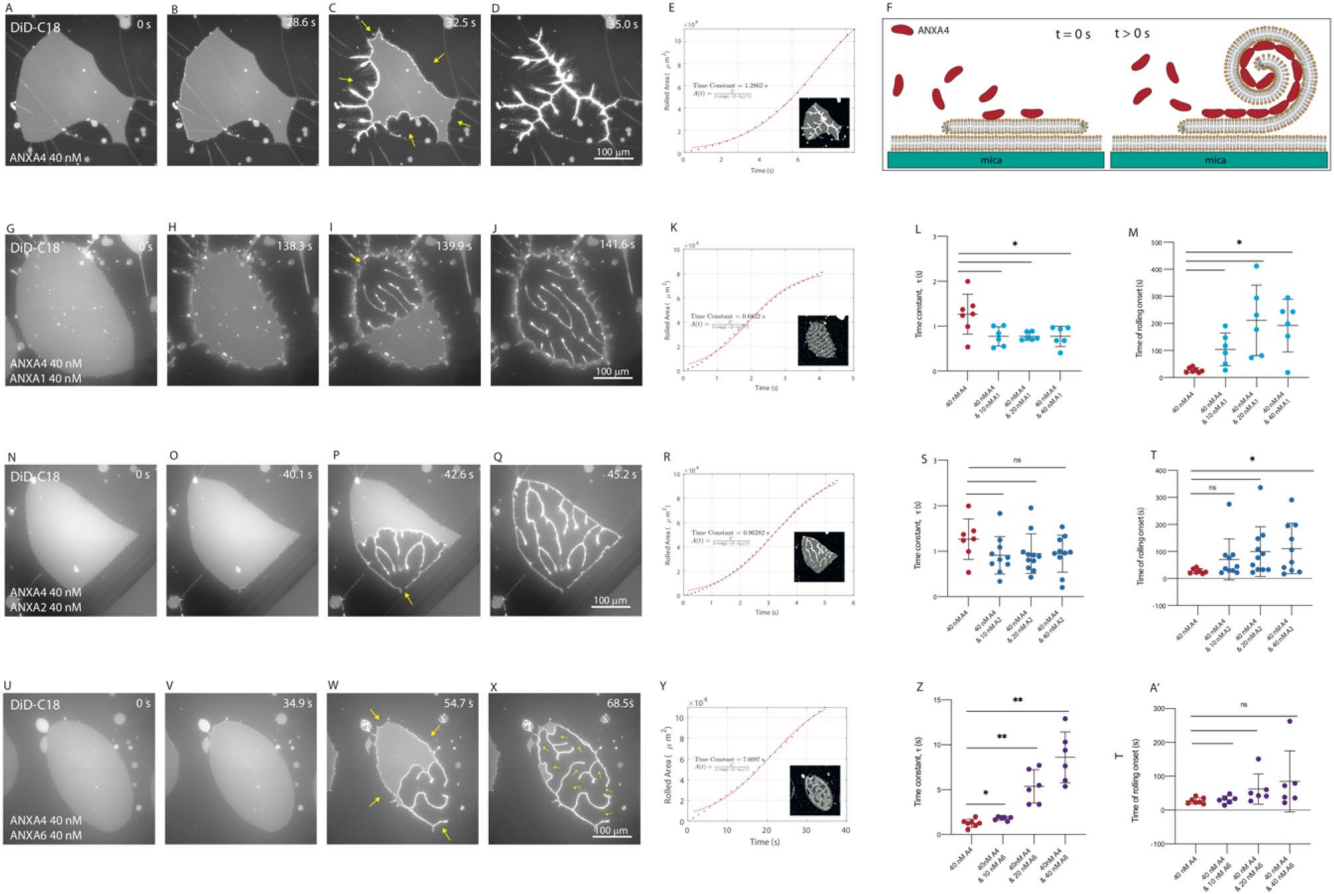
Membrane patch rolling induced by mixtures of ANXA4 and the crosslinking annexins. Response of non-vesicular membrane patches with open edges (DOPC/DOPS, 9:1 molar ratio) to exposure to annexins: 40 nM ANXA4 (**A**–**D**) induced complete, multidirectional roll up, initiated from points on the free edges (arrows, **C**). Quantification of the incremental area reduction between image frames is shown graphically (**E**) where alternating dark and light grey bands show area reduction during rolling (inset, **E**). Plotted as cumulative rolled area (blue circles, **E**) the rolling time constant, τ, was derived from a fit to a logistic function (red line, **E**). Schematic of ANXA4 induced membrane rolling of a membrane patch with open edges resting on a primary supported membrane (**F**); 40 nM ANXA4 and 40 nM ANXA1 (**G**–**J**) induced complete, unidirectional roll up from a single point (arrow, **H**) after patch edges were bound to the supporting membrane, with membrane stretching from edges causing corrugations (**H**–**J**). 40 nM ANXA4 and 40 nM ANXA2 (**N**–**Q**) induced complete, unidirectional roll up from a single point (arrow, **O**) after patch edges were bound to the supporting membrane. 40 nM ANXA4 and 40 nM ANXA6 (**U**–**X**). Multidirectional rolling, initiated from points on the free edge (arrows, **V**), followed by binding of rolled patch edges to the supporting membrane and unidirectional rolling continued from a single point for each rolling segment (**W**). Quantification of the incremental area reduction between image frames is shown graphically (inset) for mixtures of 40 nM ANXA4 with 40 nM ANXA1 (**K**), ANXA2 (**R**) and ANXA6 (**Y**). Rolling time constants for 40 nM ANXA4 and 10, 20 and 40 nM ANXA1 (light blue circles, **L**) are significantly shorter (Welch’s *t* test, *P < 0.05) than 40 nM ANXA4 (red circles, **L**). The time of rolling onset (light blue circles, **M**) is significantly longer (*) than 40 nM ANXA4 (red circles, M); Rolling time constants for 40 nM ANXA4 and 10, 20 and 40 nM ANXA2 (dark blue circles, S) are similar to 40 nM ANXA4 (red circles, S). The time of rolling onset for 40 nM ANXA4 and 20 nM ANXA2 and 40 nM ANXA4 and 40 nM ANXA2 (dark blue circles, T) is significantly longer (*) than 40 nM ANXA4 (red circles, T), but not at 40 nM ANXA4 and 10 nM ANXA2 (dark blue circles, T); Rolling time constants for 40 nM ANXA4 and 10 nM ANXA6, 40 nM ANXA4 and 20 nM ANXA6 and ANXA4 and 20 nM ANXA6 (purple circles, Z) are significantly longer (*) than for 40 nM ANXA4 (red circles, Z). The time of rolling onset for 40 nM ANXA4 and 10, 20 and 40 nM ANXA6 (purple circles, **A**′) was not significantly greater (*) than 40 nM ANXA4 (red circles, **A**′). Images prepared using NIS elements, version 5.10 (https://www.microscope.healthcare.nikon.com/products/software/nis-elements).

To study the effects of the presence of the crosslinkers (ANXA1, ANXA2, ANX6) on the curvature induction of ANXA4, the response of non-vesicular membrane patches with open edges (DOPC/DOPS, 9:1 molar ratio, 0.05% DiD) to addition of 40 nM ANXA4 alone or in combination with 10, 20 and 40 nM of crosslinking annexins was observed using time-lapse epi-fluorescence microscopy. Proteins were added from more concentrated stock to the fluid cell such that the final concentrations were as stated. Recombinant ANXA4 was added to membrane patches to serve as a comparison for the ANXA4 and crosslinker mixtures, as previously reported^6,14,15^, ANXA4 induced complete roll up, initiated from points on the free edges (Fig. 1A–D). Quantification of the incremental area reduction between image frames is shown graphically (E). By fitting to a logistic function (red line, E), to cumulative rolled area (blue circles, E), the rolling time constant, τ, was derived. These data appear in the scatter plots (Fig. 1L,S,Z) comparing ANXA4 rolling time (red circles) with the ANXA4/crosslinker mixtures. The graphic inset in the plot (E) shows alternating dark and light grey bands corresponding to area reduction during rolling (inset, E). Multidirectional rolling initiating from the points of rolling on the membrane patch edge (arrows, C). The process of membrane rolling by ANXA4 alone is shown schematically in Fig. 1F. Annexin mediated rolling, as first reported in Boye et al*.*^6^ results when annexin monomers bind to the edge of the membrane patch and trimerize, inducing a spontaneous curvature, mediated by the curved disc shape of the protein binding region. Consequently, bilayer rolling becomes energetically favorable compared to the flat initial state. Line profiles of AFM images were used to verify the presence of membrane rolls.

ANXA4 (40 nM) and ANXA1 (10, 20, 40 nM) were added to membrane patches in a fluid cell. Fluorescence data was analysed to determine tau and the time of rolling onset. Images from representative experiments (those with the rolling time constant, tau, closest to the mean) for all ANXA1 concentrations are in SI Fig. 1. The representative experiment for 40 nM ANXA4 and 40 nM ANXA1 is in Fig. 1G–J. At all concentrations of ANXA1, the membrane rolling process due to ANXA4 was altered by the presence of ANXA1. After a statistically significant delay in time of rolling onset (Fig. 1M), when compared with the data set obtained at 40 nM ANXA4, unidirectional rolling occurred from a single point (arrow, I) more significantly more rapidly than patches exposed to 40 nM ANXA4 (Fig. 1L). The delay in rolling onset and increased speed of rolling is due to binding of membrane patches edges to the supporting bilayer. The force of curvature induction by ANXA4 on the membrane to competes with the binding of the membrane patch edges to the supporting bilayer. The evidence that this is taking place is the presence of membrane puckering (Fig. 1H–J) as the membrane stretches. Once rolling is triggered by the release of the upper membrane at a point location (yellow arrow, Fig. 1I), rolling takes place rapidly due to the membrane curvature tension that has been accumulated by the binding of ANXA4. With increasing concentration of ANXA1, the degree of puckering at the membrane edges is increased, as is the time of rolling onset (though not significantly so), though the rolling times are similar for all conditions. Upon completion of membrane rolling, the membrane patch edges remain bound to the supporting bilayer (Fig. 1J).

Images from representative experiments with ANXA4 (40 nM) and ANXA2 (10, 20, 40 nM) are in SI Fig. 2. The representative experiment for 40 nM ANXA4 and 40 nM ANXA2 is in Fig. 1N–Q. At 10 nM ANXA2, the membrane patch edges are bound to the supporting membrane prior to onset of membrane patch rolling. At all concentrations of ANXA2, some patches showed multidirectional rolling from several points at the membrane edge (10 nM, 2/7; 20 nM, 1/10; 40 nM, 1/10). In the remaining experiments, edges of patches were bound to the supporting bilayer such that they remained visible after rolling completion (Fig. 1Q). Complete, unidirectional roll up membrane patches from a single point (arrow, P) then occurred. There was a significant delay in the onset of membrane rolling for 20 nM and 40 nM ANXA2 (Fig. 1T), though only in some experiments was puckering or stretching evident, therefore the rolling time constants for all ANXA2 concentrations were similar to those of ANXA4 alone (Fig. 1S). That the rolling time constants obtained in experiments with ANXA4 and ANXA1 mixtures were reduced relative to ANXA4 alone, points to an increased efficiency of binding at the membrane patch edge for ANXA1 relative to ANXA2. In both cases, while they delay the onset of rolling, ANXA1 and ANXA2 do not interfere with the capacity of ANXA4 to induce curvature in membranes.

Images from representative experiments with ANXA4 (40 nM) and ANXA6 (10, 20, 40 nM) are in SI Fig. 3. The response of a membrane patch to 40 nM ANXA4 and 40 nM ANXA6 is shown in Fig. 1U–X. Unlike experiments with ANXA1 and ANXA2, the rolling times for ANXA4 and ANXA6 mixtures increased significantly with increasing crosslinker concentration (Fig. 1Z) suggesting that either alone or in combination, competitive binding of ANXA4 and ANXA6 on the membrane patch surface reduces curvature induction or significant ANAX6 crosslinking hinders membrane rolling. To test the idea that displacement of ANXA4 by ANXA6 on the membrane patch results in longer rolling times, the response of membrane patches to 20 nM ANXA4 was observed (SI Fig. 4). In these experiments, the rolling time constant was significantly longer than for 40 nM ANXA4 (*P < 0.05) supporting the hypothesis that competition for membrane binding between ANXA4 and ANXA6 would result in lower rolling time constants.

While the onset of rolling for ANXA4 and ANXA6 mixtures was somewhat delayed, it was not significantly so at any concentration (Fig. 1A′) reflecting the fact that unlike in experiments with ANXA1 and ANXA2, the entire membrane patch edge is not bound to the supporting membrane. At all concentrations, multidirectional rolling begins from several points on the membrane edge (arrows, Fig. 1W) but is hindered, as segments of the newly revealed membrane edge appear to be bound to the supporting membrane. Rolling continues in a somewhat stepwise fashion, segment by segment, each with its own directionality (arrows, Fig. 1X). The experiment with the rolling time constant, tau, closest to the mean for all to 40 nM ANXA4 and 40 nM ANXA6 (Fig. 1U–X) also displays a feature that has not been observed in previously published rolling experiments^6,14,15^, incomplete rolling, which occurs in 1/6 experiments at 20 nM ANXA6 and 2/6 experiments at 40 nM ANXA6. This phenomenon will be discussed in detail in the next section.

### Incomplete rolling of membrane patches in the presence of ANXA4 and ANXA6 mixtures

**Figure 2:**
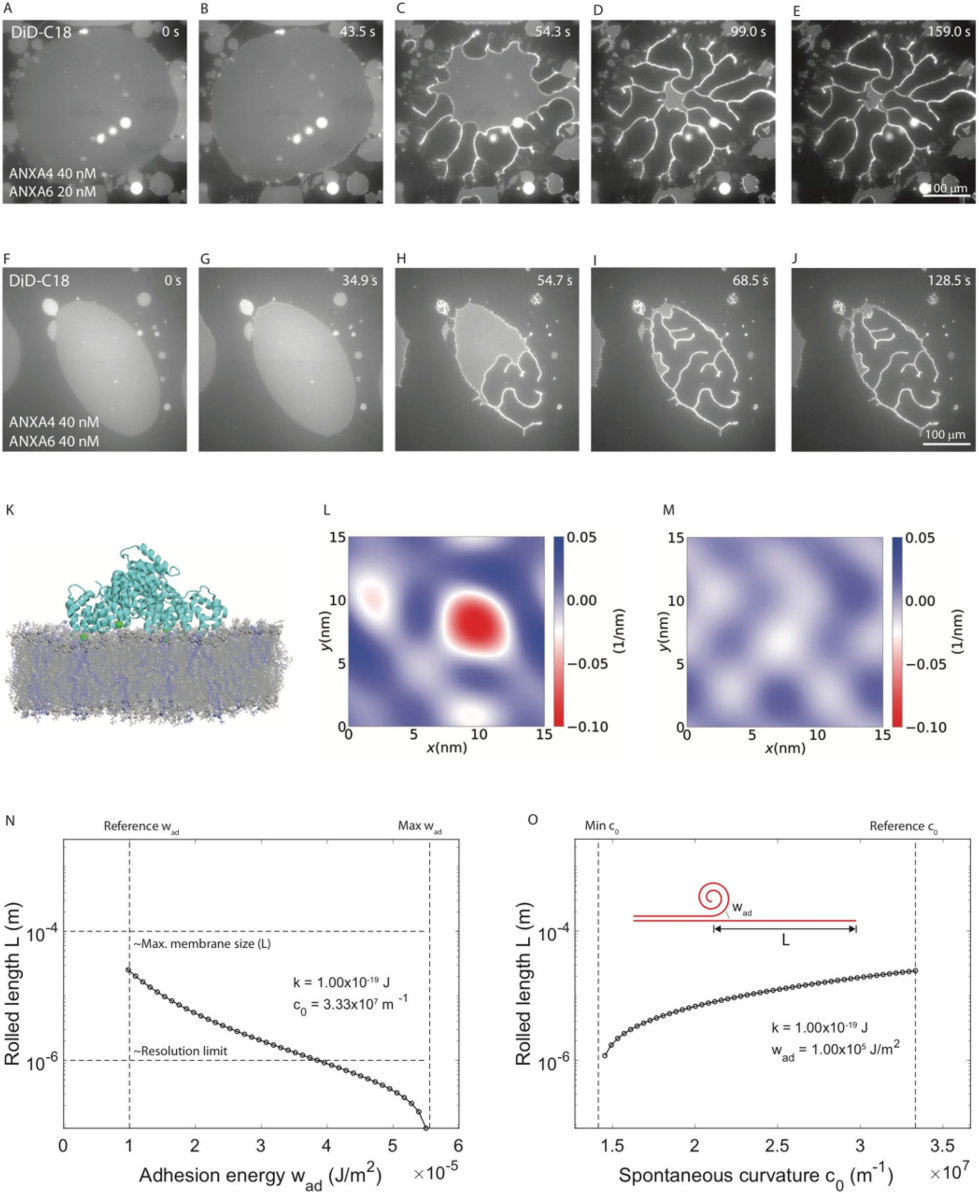
Incomplete rolling of membrane patches in the presence of ANXA4 and ANXA6 mixtures. Response of non-vesicular membrane patches with open edges (DOPC/DOPS, 9:1 molar ratio) to exposure to a mixture of 40 nM ANXA4 with 20 nM (**A**–**E**) and 40 nM (**F**–**J**) ANXA6. Molecular dynamics simulation of ANXA6 with membrane. (**K**) Final simulation snapshot. POPC lipids are shown in grey and POPS lipids are in blue. Ca^2+^ ions are shown as green spheres (**L**) Curvature profile for a surface passing through the center of the membrane with ANXA6 (**M**) Curvature profile for a surface passing through the center of the membrane without ANXA6. Theoretical modelling of the rolled membrane length L (**N**,**O**). Change in the rolled length *L* as a function of an increase in the adhesion energy w_ad_ relative to the reference value (**N**). Change in the rolled length *L* as a function of a reduction in spontaneous curvature c_0_ relative to the reference value (**O**). Images prepared using NIS elements, version 5.10 (https://www.microscope.healthcare.nikon.com/products/software/nis-elements) and Visual Molecular Dynamics, version 1.9.3 (https://www.ks.uiuc.edu/Research/vmd/).

As discussed in the previous section, the rolling time constants for ANXA4 (40 nM) and ANXA6 (10, 20, 40 nM) increase with increasing ANXA6 concentration suggesting that competitive binding of ANXA4 and ANXA6 on the membrane patch surface results in less curvature induction and/or significant crosslinking slows down rolling dynamics. At higher concentrations of ANXA6 (20 nM and 40 nM), in 3/12 experiments, a difference in membrane rolling occurs where the membrane patch rolling is incomplete. Representative experiments at each concentration are shown in Fig. 2 (20 nM, A–E; 40 nM, F–J). As in the previous section, the patch at 0 s, the onset of rolling, midway and rolling completion are shown. An extra frame is included that shows in both experiments, sections of membrane patch that remain unrolled, 60 s after rolling has completed. This has not occurred in any membrane patch experiments in this and other publications^6,14,15^ and may be due to two physical effects, competition for binding on membrane patches of ANXA6 with ANXA4 and ANXA6 membrane crosslinking of adjacent membranes.

The rolling time constants obtained in membrane patch experiments with 20 nM ANXA4 were significantly longer than for 40 nM ANXA4 (SI Fig. 4) supporting the hypothesis that competition for membrane binding between ANXA4 and ANXA6 would result in lower rolling time constants. To further investigate if competitive binding may play a role in both incomplete rolling and the increased rolling time constants for ANXA4 and ANXA6 mixtures with increasing ANXA6 concentration, all atom molecular dynamics simulations of ANXA6 in the proximity of the lipid bilayer were performed (Fig. 2K). Like other annexins, ANXA6 binds to the lipid bilayer mediated by calcium ions and induces negative curvature. One of the core domains induces higher curvature compared to the other (Fig. 2L), possibly due to the difference in the number of calcium ions bridging the two domains to the membrane. The curvature profile for a surface passing through the center of the membrane without ANXA6 was determined as a control (Fig. 2M).The overall mean curvature induced under the protein ANXA6, averaged over three replicas, is 0.0083 ± 0.0014 nm^−1^, which is significantly lower than the mean curvature induced under the ANXA4 trimer (0.0240 ± 0.0002 nm^−1^)^34^. These data demonstrate that per unit area, ANXA6 induces less curvature than ANXA4 trimers, in good agreement with the idea that competitive binding of ANXA4 and ANXA6 is taking place.

To investigate if ANXA6 crosslinking may play a role in both incomplete rolling and the increased rolling time constants for ANXA4 and ANXA6 mixtures with increasing ANXA6 concentration, the rolled length, L, was calculated as a function of adhesion energy, w_ad_ (Fig. 2N) and spontaneous curvature c_0_ (Fig. 2O) using the biophysical model for membrane rolling that was developed in Boye et al*.*^6^. Briefly, the model describes the rolling of a membrane as starting from the free edge and subject to spontaneous curvature, c_0_, originating from asymmetric binding of annexins and subject to crosslinking between the membrane patch and the underlying primary membrane. The energy decrease from the flat initial state to the rolled final state determines the extent of rolling and the final rolled membrane length, *L,* as indicated in Fig. 2N. The curvature elastic energy of the membrane is described using the Helfrich expression^35^. The rolling model was implemented with the mean curvature elastic modulus, k = 1.0 × 10^–19^ J, spontaneous curvature, c_0_ = 3.33 × 10^7^ m^−1^, and adhesion energy, w_ad_ = 1.0 × 10^5^ J/m^2^*.* Figure 2O shows that increasing the adhesion, thereby modeling crosslinking by ANXA6, results in the rolled length *L* being strongly reduced. For example, increasing w_ad_ by a factor ~ 3.5 reduces the rolled length to around the resolution limit ~ 1 μm (i.e. no rolling). Similarly, Fig. 2N shows that a reduction in the spontaneous curvature, c_0_, modeling the lower density of ANXA4 when ANXA6 binds, also leads to a reduction in the rolled length. A reduction of the spontaneous curvature by roughly 50% leads to the rolled length being reduced to the resolution limit ~ 1 μm (no rolling). To summarize, the model describes that membrane crosslinking and/or reduced spontaneous curvature can lead to a reduction in the rolled membrane length in agreement with the observations made for ANXA4 in combination with ANXA6. Taken together, our molecular dynamics simulations and our model for rolling length suggest that both of these physical effects may be present in the system, though we cannot speculate as to their relative contributions to the data.

### Localization of crosslinkers during membrane patch rolling

**Figure 3:**
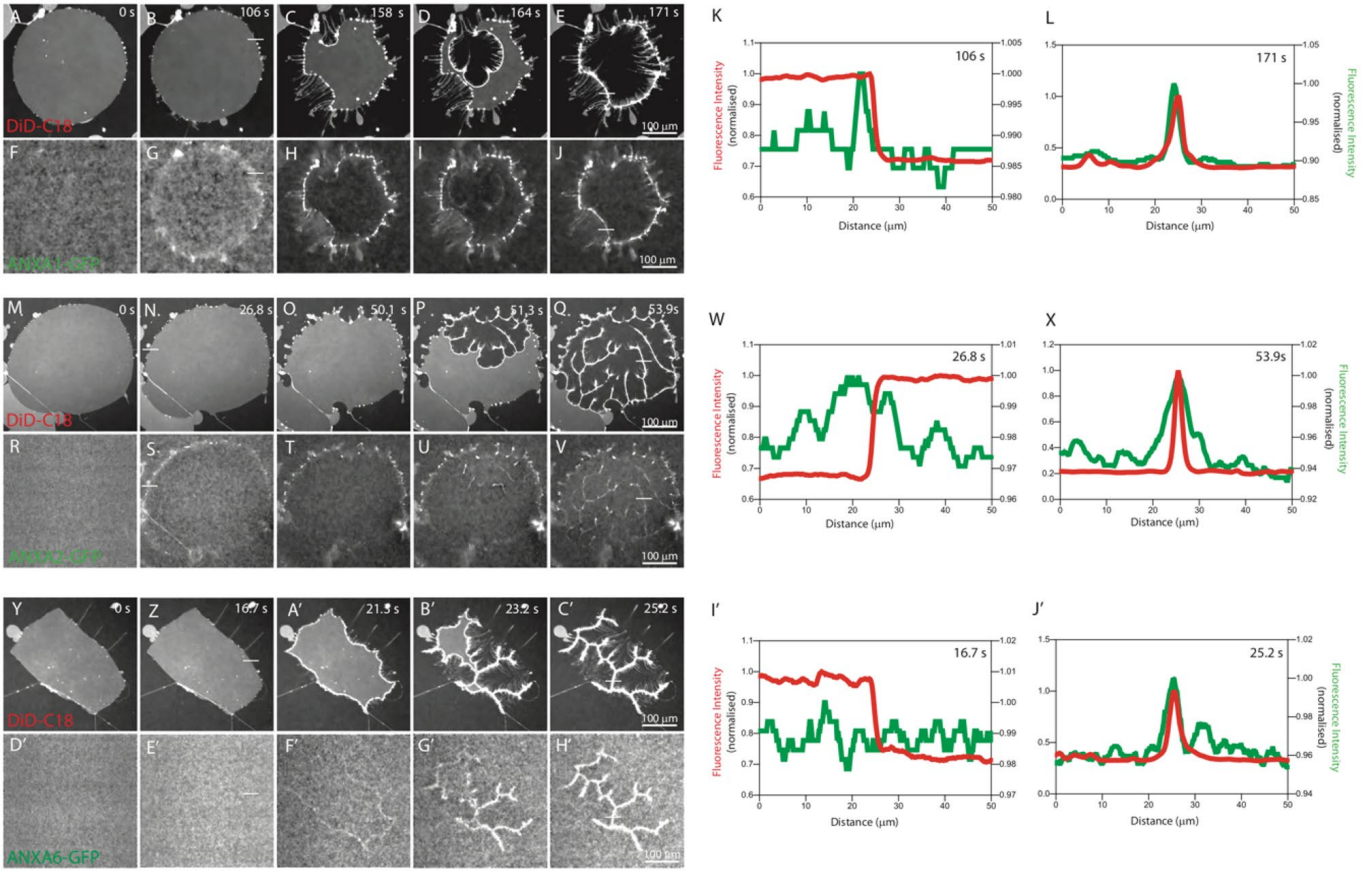
Localization of crosslinkers during membrane patch rolling. Response of non-vesicular membrane patches with open edges (DOPC/DOPS, 9:1 molar ratio) to exposure to annexins when 1:1 annexin mixtures of ANXA4 and ANXA1-GFP (DiD, **A**–**E**; GFP, **F**–**J**), ANXA4 and ANXA2-GFP (DiD, **M**–**Q**; GFP, **R**–**V**) and ANXA4 and ANXA6-GFP (DiD, **Y**–**C**′; GFP, **D**′–**H**′)) are added to non-vesicular membrane patches with open edges (DOPC/DOPS, 9:1 molar ratio). Cross-sections taken on rolling onset (white line ANXA1, **B**,**G**, ANXA2, **N**,**S**, ANXA6, **Z**,**E**′) show that maximum ANXA-GFP fluorescence intensity (green line) coincides with the membrane patch edge as defined by DiD fluorescence (red line) for ANXA1 (**K**) and ANXA2 (**W**) and has no maximum for ANXA6 (**I**′).Cross-sections taken on rollup completion (white line ANXA1, **E**, ANXA2, **Q**, ANXA6, **C**′) show that maximum ANXA-GFP fluorescence intensity (green line) coincides with the rolled membrane as defined by DiD fluorescence (red line) for ANXA1 (**L**), ANXA2 (**X**) and ANXA6 (**J**′). Images prepared using NIS elements, version 5.10 (https://www.microscope.healthcare.nikon.com/products/software/nis-elements).

To determine if the localization of crosslinkers reflects the different rolling morphologies, the response of non-vesicular membrane patches with open edges (DOPC/DOPS, 9:1 molar ratio, 0.05% DiD) to the addition of 40 nM ANXA4 in combination 40 nM of GFP labelled crosslinking annexin was observed using confocal microscopy. Proteins were added to the fluid cell from concentrated stock solutions such that the final concentrations were as stated. The typical response of a membrane patch to 40 nM ANXA4 and 40 nM ANXA1-GFP and ANXA4 and ANXA2-GFP can be seen in DiD (ANXA1 GFP, Fig. 3A–E; ANXA2-GFP, Fig. 3M–Q) and GFP channels ((ANXA1 GFP, Fig. 3F–J; ANXA2-GFP, Fig. 3R–V).

The images at 106 s for ANXA1-GFP (DiD, Fig. 3B; GFP, Fig. 3G) and 26.8 s for ANXA2-GFP (DiD, Fig. 3N; GFP, Fig. 3S) show the membrane patch just prior to the onset of membrane rolling as identified in the DiD channel. The shape of the patch is not yet significantly modified by the presence of the annexins, but the GFP image indicates that crosslinkers localize to the membrane patch edges, quantified by the line profiles (white line Fig. 3B,G,N,S) that show maximal GFP fluorescence (green line, Fig. 3K,W) coincides with the membrane patch edge as indicated by the DiD fluorescence (red line, Fig. 3K,W). As rolling proceeds, GFP fluorescence remains localized to the membrane edges, including newly revealed edges created by membrane rolling (ANXA1-GF, Fig. 3H,I; ANXA2-GFP, Fig. 3T–U). Once membrane rolling is complete, maximal GFP fluorescence (green line Fig. 3L,X) coincides with membrane rolls as indicated by DiD fluorescence (red line Fig. 3L,X).

The typical response of a membrane patch to 40 nM ANXA4 and 40 nM ANXA6-GFP can be seen in DiD (Fig. 3Y–C′) and GFP channels (Fig. 3D′–H′).

Confocal observation of membrane patches after the addition of 40 nM ANXA4 and 40 nM ANXA6 revealed a significant difference in crosslinker localization.

The images at 16.7 s for ANXA6-GFP (DiD, Fig. 3Z; GFP, Fig. 3E′) show the membrane patch just prior to the onset of membrane rolling as identified in the DiD channel. Unlike in experiments with ANXA1-GFP and ANXA2-GFP, ANXA6-GFP does not localize to membrane patch edges prior to the onset of membrane rolling as quantified by the line profile (white line Fig. 3Z,E′) that show constant GFP fluorescence (green line, Fig. 3I′) coincides with the membrane patch edge as indicated by the DiD fluorescence (red line, Fig. 3I′). As rolling proceeds, ANXA6-GFP localizes to newly revealed edges created by membrane rolling (Fig. 3F′,G′). Upon completion of rolling, maximal GFP fluorescence (green line Fig. 3J′) coincides with membrane rolls as indicated by DiD fluorescence (red line Fig. 3J′) as with ANXA1-GFP and ANXA2-GFP.

These observations are in good agreement with the rolling analyses in Fig. 1. Both ANXA1 and ANXA2 have been shown to localize at the membrane patch edge prior to rolling (Fig. 3K,W respectively) and cause a delay in the onset of membrane rolling (Fig. 1M,T respectively). Compared to ANXA4 alone, the rolling time constants were either reduced (ANXA1) or unaltered (ANXA2), suggesting that ANXA1 and ANXA2 do not significantly interfere with ANXA4 curvature induction although occupying part of the membrane area. ANXA6 does not localize to at the membrane patch edge prior to rolling (Fig. 3I′) and causes no significant delay in the time of rolling onset (Fig. 1A′) but does increase the rolling time constant obtained with increasing ANXA6 concentration, suggesting it does interfere with ANXA4 curvature induction.

### Atomic force microscopy characterization of the mixing of ANXA4 and the crosslinking annexins

**Figure 4:**
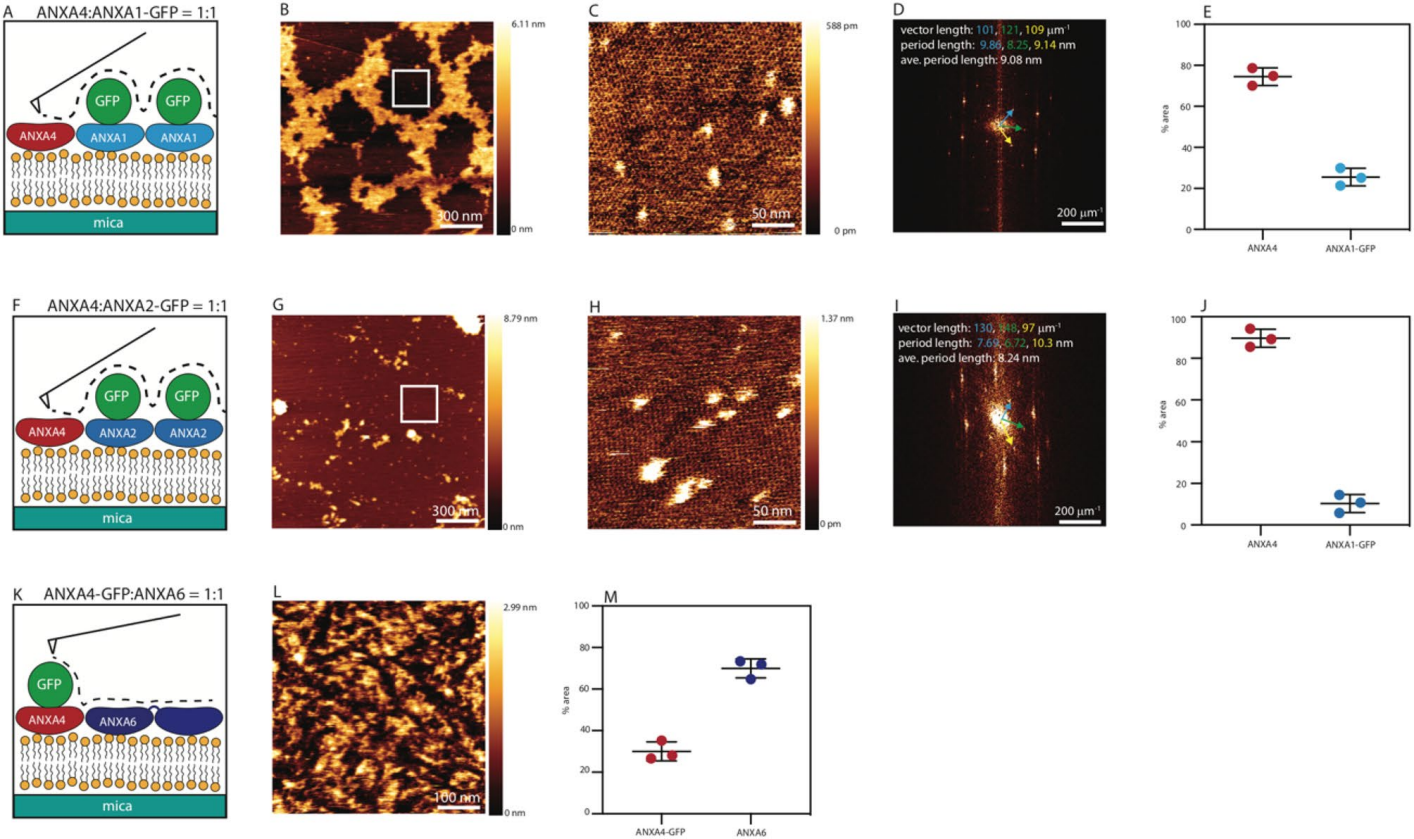
Atomic force microscopy characterization of the mixing of ANXA4 and the crosslinking annexins. Schematic representation of AFM imaging of proteins structures that form when 1:1 annexin mixtures (ANXA4 and ANXA1-GFP (**A**), ANXA4 and ANXA2-GFP (**E**) and ANXA4-GFP and ANXA6 (**I**)) are added to DOPC/DOPS (1:1 molar ratio) supported lipid bilayers where the presence of the GFP label on provides a height differential between the two proteins. 1.5 μm scan on a mixture of ANXA1-GFP and ANXA4 indicates that the proteins do not mix (**B**). 250 nm scan (white square, **B**) allows ANXA4 trimers to be resolved (**C**). Fourier transform of (**C**) indicates a crystalline structure (**D**). Lattice vectors in Fourier space are indicated by arrows with corresponding vector length and period lengths noted in the same color. 1.5 μm AFM image of the protein structure that forms when a 1:1 mixture of ANXA4 and ANXA2-GFP is added to a supported lipid bilayer (shown schematically, **E**) (**F**). 250 nm scan (white square, **F**) allows ANXA4 trimers to be resolved (**G**). Fourier transform of (**G**) indicates a crystalline structure (**D**). Lattice vectors in Fourier space are indicated by arrows with corresponding vector length and period lengths noted in the same color. 500 nm AFM image of the protein structure that forms when a 1:1 mixture of ANXA4-GFP and ANXA6 is added to a supported lipid bilayer (shown schematically, **I**) (**J**). 250 nm image (**L**). Quantification of area occupied by ANXA4-GFP (red circles) and ANXA6 (purple circles) in a 1:1 mixture in 500 nm scans (N = 20, from 3 independent experiments) indicates that ANXA4-GFP occupies 30% of scan area and ANXA6 occupies 70% of scan area (**M**). Images prepared using JPK Data Processing Software, version 7.01.145 (https://www.bruker.com/de/products-and-solutions/microscopes/bioafm/jpk-nanowizard-4-xp-bioscience.html).

To study how ANXA4 mixes with the crosslinkers, AFM imaging was performed on structures that formed when ANXA4 and crosslinker protein mixtures, contained one GFP labelled protein, were added to DOPC/DOPS (1:1 M ratio) SLBs. The presence of the 27 kDa GFP label on either 36 kDa ANXA1, ANXA2 or ANXA4 served as a topographical marker, providing a height differential between the two protein species being investigated, shown schematically for ANXA4 and ANXA1-GFP (Fig. 4A), ANXA4 and ANXA2-GFP (Fig. 4F) and ANXA6 and ANXA4-GFP (Fig. 4K). All data presented here come from at least 6 scans from 3 independent experiments.

The 1.5 µm × 1.5 µm AFM image obtained by scanning on the surface of the protein structures that form when ANXA4 and ANXA1-GFP is added to the fluid cell (Fig. 4B), indicates that two distinct height domains form, the lower (brown) region corresponding to unlabelled ANXA4 and the higher (yellow) region corresponding ANXA1-GFP. The presence of ANXA4 in the topographical low of the image was confirmed by performing a 250 nm × 250 nm scan (Fig. 4C, scan region indicated by white square in Fig. 4B) where individual trimers (~ 10 nm diameter) were resolved. A Fourier transform allows the average periodic length of the image to be determined. Trimers in the ANXA4 250 nm image (Fig. 4C) close pack in a p3 lattice and the Fourier transform (Fig. 4D) confirms crystallinity. The average period length of 9.08 nm corresponds to the size of the crystal’s constituent trimers.

The formation of these structures can be captured by successive AFM scans on the same membrane area as soon as the protein mixture is added (SI Fig. 5B–E). In this image series, the first apparent topographical change is caused by the formation of ANXA4 crystals. At later time points, another topographical change occurs with the binding of ANXA1-GFP in the gaps left by the ANXA4 crystals, indicating that on a flat membrane, ANXA4 and ANXA1GFP do not mix. The % area occupied by ANXA4 and ANXA1-GFP in 6 scans from 3 independent experiments is shown in Fig. 4E at 75.45 and 24.55 respectively.

The protein structures formed when 40 nM ANXA4 and 40 nM ANXA2-GFP (Fig. 4G) are added to the fluid cell are similar to those formed by ANXA4 and ANXA1-GFP with two distinct height domains forming, the lower (brown) region corresponding to unlabelled ANXA4 and the higher (yellow) region corresponding ANXA2-GFP. Trimers were resolved in a 250 nm × 250 nm scan (Fig. 4H, scan region indicated by white square in Fig. 4G). The average periodic length, 8.24 nm, obtained by performing a Fourier transform (Fig. 4I) is indicative of crystalline close packing of trimers. As with ANXA4 and ANXA1-GFP, the formation process for the ANXA4 and ANXA2-GFP protein structure (SI Fig. 5G–J) indicates that the ANXA4 crystals form prior to the binding of ANXA2-GFP and these proteins do not mix with percentage scan area of ANXA4 and A2GFP at 9.499% and 90.50% respectively (Fig. 4J, data obtained from 6 scans from 3 independent experiments). Significantly less scan area is occupied by ANXA2-GFP (Fig. 4G) than ANXA1-GFP (Fig. 4B) when mixed with ANXA4 (SI Fig. 6, *P < 0.05).

AFM scans of the structures formed when 40 nM ANXA4-GFP and 40 nM ANXA6 (Fig. 4L) are added to the SLBs are similar to those formed by ANXA4 and ANXA5 reported previously^15^. The topographical variation in the scan is indicative of protein mixing with the % area occupied by each species (ANXA4-GFP 29.45 vs ANXA6 70.55) suggestive of similar binding affinity given the size differential between the two proteins (ANXA6 contains two annexin core domains versus one for ANXA4) (Fig. 4M).

The differential mixing of the crosslinkers with ANXA4 are in good agreement with the data obtained in membrane rolling and crosslinker localization experiments. Both ANXA1 and ANXA2 do not readily mix with ANXA4. The time series of AFM images collected on ANXA4 and crosslinkers mixtures on supported lipid bilayers demonstrates that the structures formed in Fig. 4B (ANXA1) and 4G (ANXA2) form first by the crystallization of ANXA4, followed by binding of the GFP labelled proteins. In the final structures, significantly less ANXA2-GFP (Fig. 4G) was bound to the membrane than ANXA1-GFP (Fig. 4B) suggesting that it has a lower membrane binding affinity than ANXA1-GFP in the presence of ANXA4. The localization of ANXA1 and ANXA2 to the membrane patch edge and lack of hindrance of ANXA4 mediated patch rolling suggests preferential binding of ANXA1 and ANXA2 to regions of high curvature rather than the low curvature membrane patch surface, analogous to the flat supported lipid bilayers used for AFM experiments. ANXA6 however, while showing no affinity for high curvature membrane patch edges, mixes readily on a flat surface with ANXA4. This is in good agreement with membrane patch experiments with ANXA4 and ANXA6 mixtures that demonstrate that ANXA6 hinders ANXA4 induced curvature.

### Hindrance of membrane patch unrolling by crosslinking annexins

**Figure 5:**
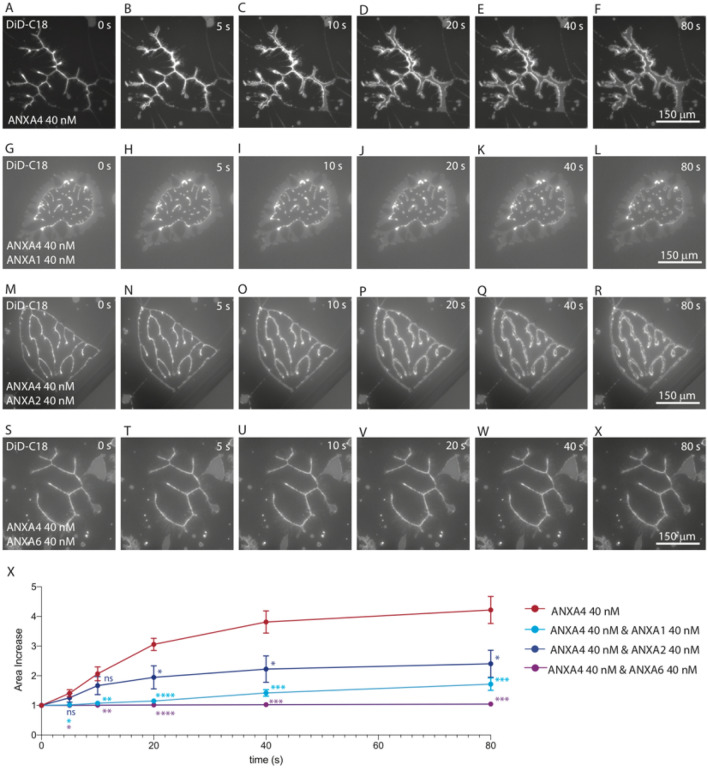
Hindrance of membrane patch unrolling by crosslinking annexins. Time course of membrane patches unrolling from maximally rolled state for ANXA4 (**A**–**F**), ANXA4 and ANXA1 (**G**–**L**), ANXA4 and ANXA2 (**M**–**R**) and ANXA4 and ANXA6 (**S**–**X**). Relative area increase over time shown for ANXA4 (red), ANXA4 and ANXA1 (light blue), ANXA4 and ANXA2 (dark blue) and ANXA4 and ANXA6 (purple) (**X**). Results of Welch’s *t* tests comparing the relative area increase of membrane patches in the presence of ANXA4 and crosslinker mixtures relative to ANXA4 alone are indicated where *P < 0.05. Images prepared using NIS elements, version 5.10 (https://www.microscope.healthcare.nikon.com/products/software/nis-elements).

The rolling time constants used to characterize cooperative rolling result from ANXA4 binding to a membrane patch are obtained by analyzing the images that are collected from the beginning of the experiment (prior to addition of protein) up until the membrane patch is maximally rolled up. After this time point, the membrane patch partially unrolls to a lower energy state as can be seen in images taken from a time course of a representative experiment with 40 nM ANXA4 (Fig. 5A–F). The most likely explanation for unrolling is that after roll completion, Annexins start to bind to the outer roll surface thus cancelling the spontaneous curvature and favoring a flatter membrane shape. However, unrolling will be hindered by membrane crosslinking inside rolls meaning that restricted unrolling is a convenient indicator of crosslinking efficiency.

Membrane patches exposed to ANXA4 and crosslinker mixtures were observed after maximal roll up. The relative area increase was calculated for images of patches unrolling from a maximally rolled state for 80 s. Representative experiments for 40 nM ANXA4 and 40 nM crosslinker can be seen in Fig. 5: ANXA1, G-L; ANXA2, M-R; and ANXA6, S-X. The relative area increase over time for ANXA4 (red), ANXA4 and ANXA1 (light blue), ANXA4 and ANXA2 (dark blue) and ANXA4 and ANXA6 (purple) is shown in Fig. 5X. The most significant contrast to ANXA4 alone (Fig. 5X, red circles) is that of the ANXA4 and ANXA6 mixture (Fig. 5X, purple circles) where negligible area change occurs throughout the observation period, meaning unrolling is entirely prevented by the presence of the crosslinker. Between these two extremes, ANXA4 and ANXA1 (Fig. 5X, light blue circles) with significant hindrance of membrane rolling throughout the observation period and ANXA4 and ANXA2 (Fig. 5X dark blue circles) with significant hindrance after 20 s. Results of Welch’s *t* tests comparing relative area increase of the membrane patches in the presence of ANXA4 and crosslinker mixtures to ANXA4 alone are indicated in Fig. 5X where *P < 0.05.

The differential capacity of the crosslinkers to hinder membrane unrolling after maximal membrane patch roll up is in good agreement with the results of mixing experiments. ANXA6 that mixes and binds readily on the surface of membrane patches with ANXA4 results in no membrane unrolling. ANXA1 and ANXA2 that show higher affinity for high curvature membrane when added with ANXA4 have less capacity to prevent membrane unrolling. Though it is worth noting that ANXA1 is significantly better able to hinder unrolling than ANXA2 for the entire observation period. This may be due to more ANXA1 binding than ANXA2.

### Studying crosslinkers in a dynamic setting allows relative crosslinking ability to be determined

By studying crosslinkers in a dynamic setting created by ANXA4 induced curvature on membrane patches with free edges, we can characterize the relative capacities of ANXA1, ANXA2 and ANXA6 as crosslinkers. Considering this data, together with confocal microscopy localization data and AFM characterization of annexin mixing, provides new insight into the mechanism by which PMR takes place. When added in combination with ANXA4, ANXA6 mixes with ANXA4 and binds adjacent membranes to a greater extent than both ANXA1 and ANXA2. In mixing readily with ANXA4 on membrane patches, ANXA6 reduces the extent to which ANXA4 induces curvature, but does not delay or prevent it. This observation can be explained by two physical effects, competitive binding that reduces spontaneous curvature and ANXA6 crosslinking. Our molecular dynamics simulations show that per unit area, ANXA6 induces less curvature than ANXA4 trimers and our model for rolling length demonstrates that ANXA6 membrane crosslinking leads to a reduction in the rolled membrane length. Our data suggest that both of these physical effects may be present in the system, though we cannot speculate as to their relative contributions to the data. These findings support the Boye et al*.*^6^ model of PMR where ANXA6, recruited first to the wound, is required for efficient sealing of the hole via crosslinking. ANXA4, recruited after, induces out-of-plane curvature so that together, the curvature and constriction forces result in wound sealing. After recruitment, both ANXA4 and ANXA6 act in concert, therefore, the capacity of ANXA4 and ANXA6 to mix on a membrane surface is an important piece of supporting data for the Boye et al*.* model^6^. It should be noted here however, that our discussion of ANXA6 only refers to its role in PMR as it binds to membranes and therefore our findings to not contradict the self-interaction described in the ANXA6 repair cap model^21,22^.

When added in combination with ANXA4, ANXA1 and ANXA2 localize to the region near free membrane edges, delaying but not hindering ANXA4 induced curvature. ANXA1 binds more effectively to flat membranes in the presence of ANXA4 than ANXA2 and both proteins crosslink to adjacent membranes, ANXA1 to a greater extent than ANXA2. These subtle but significant differences support the idea that ANXA1 and ANXA2 have functional differences in PMR^26^. However, the data presented here pertain only to ANXA1 and ANXA2 binding and crosslinking as monomers where an important aspect of the role of ANXA1 and ANXA2 in PMR are their binding partners, S100A11 and S100A10 proteins respectively^36^. An interesting direction forward would be the inclusion of these and other relevant species, such as additional sources of membrane in future studies.

**Figure 6:**
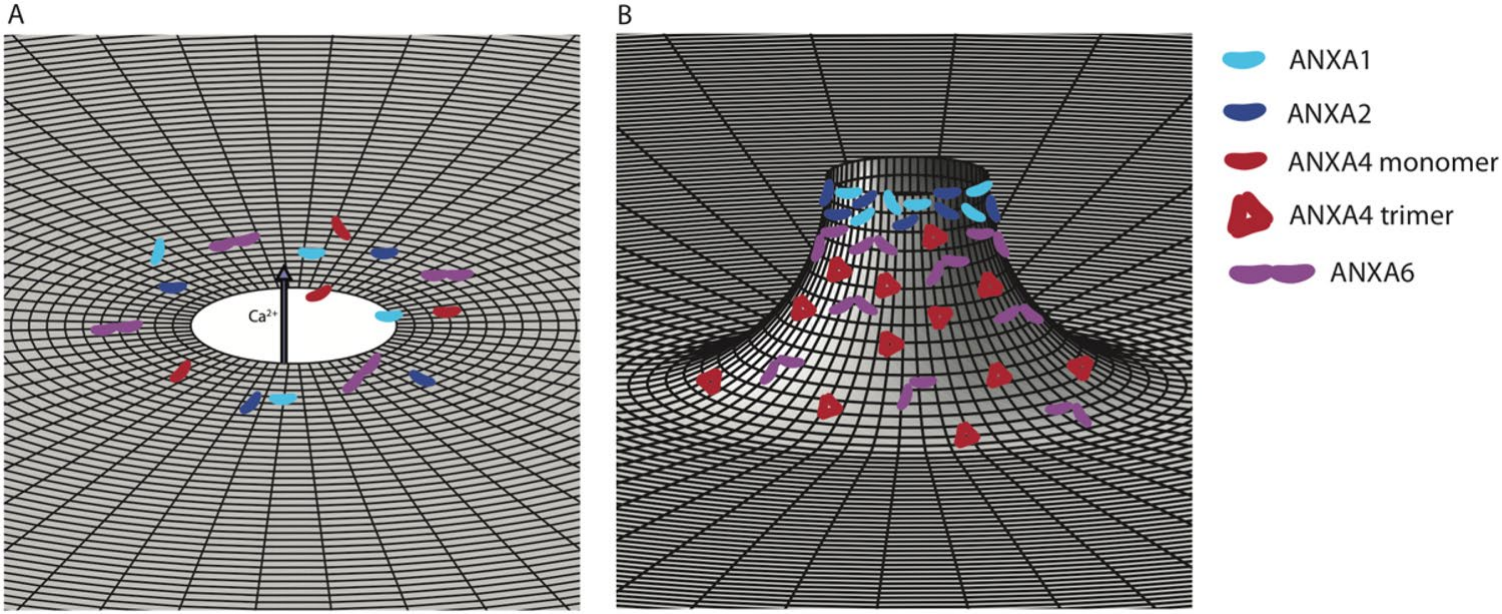
Schematic representation of PMR mediated by ANXA1, ANXA2, ANXA4 and ANXA6 action. In uninjured cells, annexins are distributed uniformly as monomers in the cytoplasm. Upon local plasma membrane injury, Ca^2+^ influx (**A**) results in recruitment of annexins to the membrane wound edges (**B**). ANXA1 and ANXA2 preferentially bind to free membrane edges (light and dark blue respectively, **B**) positioning them as sites of recruitment for binding partners involved in repair processes and for crosslinking to adjacent membranes such as intracellular vesicles that arrive at the damage site as membrane resupply. Binding as trimers, ANXA4 induces out of plane curvature (red, **B**), mixing with ANXA6 which provides constriction force to the membrane (purple, **B**) and is available to bind to sources of membrane resupply.

Taken together, these data can provide an understanding of the relative capacity of ANXA1, ANXA2 and ANXA6 to perform membrane crosslinking as shown schematically in Fig. 6. Upon influx of Ca^2+^ (Fig. 6A), cytoplasmic annexins are recruited to the membrane around the damage site. Binding as trimers, ANXA4 trimers induce curvature (red, Fig. 6B), mixing with ANXA6 which crosslinks adjacent areas of the membrane near the damage site (purple, Fig. 6B). ANXA1 and ANXA2 preferentially bind to free membrane edges (light and dark blue respectively, Fig. 6B) positioning them for crosslinking to adjacent membranes such as intracellular vesicles that arrive at the damage site as membrane resupply. At the free edge, ANXA1 and ANXA2 are also positioned as sites of recruitment for binding partners involved in repair processes. Unlike ANXA1 and ANXA2, ANXA6 shows no specific localisation and is able to crosslink adjacent membranes anywhere it binds near the damage site, which it does with greater efficiency than ANXA1 and ANXA2. Current data suggests that the relative recruitment of the annexins in living cells is species dependent, particularly for ANXA2, and as such, we cannot speculate on the order of arrival of the annexins in this study at the wound site. However, our data here allows the mechanistic model of PMR proposed by Boye et al*.*^6^ to be expanded to include ANXA1 and ANXA2 as specialist free edge membrane crosslinkers.

Studies of the annexins have been overwhelmingly performed using a single annexin, despite the recruitment of multiple annexins to damage sites in cells, raising questions of cooperativity in PMR. Hence, this investigation of the effect of the presence of the crosslinking annexins, annexin A1, A2 and A6 (ANXA1, ANXA2 and ANXA6) on the membrane curvature induction of annexin A4 (ANXA4) in model membrane systems. Using membrane patches with free edges, we have demonstrated that the curvature induction of ANXA4, a complete roll up of membrane patches, is not prevented by the presence of ANXA1, ANXA2 and ANXA6, though the speed with which ANXA4 induces membrane roll up was reduced in the presence of ANXA6. ANXA4 and ANXA6 were found to mix uniformly so this observation is likely due to two physical effects: competitive binding with ANXA4 that induces more curvature per unit area than ANXA6 and ANXA6 membrane crosslinking. In the presence of ANXA4 induced curvature, ANXA6 displays a higher capacity to crosslink adjacent membranes than ANXA1 and ANXA2 respectively that were found not to mix readily with ANXA4. Conversely, ANXA1 and ANXA2, both found to localize to membrane edges, were most effective as crosslinkers at membrane patch free edges. Taken together, these data suggest that ANXA1 and ANXA2 are specialist free edge membrane crosslinkers that act in concert with ANXA6 crosslinking and ANXA4 curvature induction, demonstrating that the interplay of curvature inducing and crosslinking annexins must be considered in mechanistic models of PMR.

## Methods

### Production and purification of recombinant proteins

Recombinant Annexins were produced and purified using previously described method^6,14^. In short, PCR amplified Annexin cDNA constructs were subcloned into the bacterial expression vector pETM11-SUMO3 (originally from EMBL Protein Expression and Purification Core Facility) with or without C-terminally tagged superfold GFP (sfGFP) and N-terminally tagged with a 6xHis-tag and a SUMO3 domain. BL21 (DE3) competent *Escherichia* *coli* cells were used as expression hosts for the production of recombinant proteins. Protein expression was induced overnight at 18 °C using Isopropyl β-d-1 thiogalactopyranoside (IPTG). Cells were harvested by centrifugation and lysed by sonication. Proteins were purified by Immobilized Metal-Affinity Chromatography (IMAC) using Ni-NTA (nickel-nitrilotriacetic acid) resins (Qiagen). The N-terminal 6xHis-tag and SUMO3 domain were cleaved off by SUMO-Specific Protease 2 (SENP2) (molar ratio 200:1) followed by dialysis overnight at 4 °C. Proteins were further purified and separated using Fast Protein Liquid Chromatography (FPLC) on a Superdex 200 size-exclusion chromatography column (SuperdexTM 200, 10/300 GL, GE Healthcare Life Sciences). Protein fractions were collected based on the UV spectra. Part of the fractions were run on a 4–15% SDS-PAGE gel and stained with coomassie (SimplyBlue™ SafeStain, Thermofisher) for verification of protein size. The Ca^2+^- and lipid-binding activities of purified recombined ANXs were confirmed as described previously^14,24^. The proteins were stored at − 80 °C until use.

### Membrane patch experiments

Mica substrates (Plano GmbH), glued to glass coverslips using a silicone elastomer (MED-6215, Nusil Technology), were cleaved immediately prior to use. A 40 μL droplet of 10 mM total lipid stock (containing DOPC (1,2-dioleoyl-sn-glycero-3-phosphocholine) and DOPS (1,2-dioleoyl-*sn*-glycero-3-phospho-l-serine), 9:1 molar ratio in methanol (hypergrade for LC–MS, Merck) containing 0.5% DiD-C18 probe (Thermo-Invitrogen)) was applied to the mica, spun on a spincoater (KW-4A, Chemat Technology) at 150*g* for 40 s and placed under vacuum in a desiccator for 10–12 h to ensure solvent evaporation. The spin coated lipid film was hydrated in 10 mM TRIS buffer (2-Amino-2-(hydroxymethyl)propane-1,3-diol), 140 mM NaCl, 2 mM Ca^2+^, pH = 7.4, and buffer exchanged > 10 times to prepare defined secondary bilayer patches resting on a continuous primary membrane. Hydrated membrane patches were cooled to 22 °C and equilibrated before experiments.

For kinetic data (Figs. 1, 2 and SI Figs. 1–3), the response of bilayer patches to the addition of annexins was monitored at 22 °C with time-lapse epi-fluorescence microscopy using a Nikon Ti2-E inverted microscope with 40 × objective (Nikon ELWD S Plan Fluor, NA = 0.6). Fluorescence excitation at 550 nm was achieved using a white light fluorescence illumination system (CoolLED, pE-300). Emission was collected from 650 nm (Nikon Cy5-A, M376571). Images were captured at 10 fps with a digital CMOS camera (ORCA-Flash 4.0 V3, Hamamatsu, 2048 × 2044 pixels) using NIS-Elements software, version 5.10 (Nikon). At least 5 experiments per condition were performed. In each experiment, annexin was added to the fluid cell from a known concentrated solution such that the final bulk concentration was 40 nM. Time-lapse sequences of membrane rolling were analysed with methods written in MATLAB (Mathworks) and described in detail elsewhere^6,14^. Briefly, incremental rolled membrane area was determined by subtraction of subsequent frames in the sequence and then binarized with a cutoff. Total rolled area as a function of time was fitted by a sigmoid function with a time constant, τ, which provided a characteristic time scale for the rolling process.

Localisation of GFP labelled crosslinkers was determined using two microscope set ups: A Nikon TE2000 inverted microscope with 40 × objective (Nikon ELWD, Plan Fluor, NA = 0.6) equipped with a switchable Xenon lamp (PolychromeV, Till Photonics GmbH, Grafeling, Germany) and a dual wavelength filter cube for imaging at 640 nm (DiD) and 488 nm (GFP). Images were recorded with an emccd camera (Sensicam em, 1004 × 1002 pixels, PCO-imaging, Kelheim, Germany) and operated with Live Aquisition software (FEI GmbH); and, a Nikon A1R confocal microscope was used with a 60 × water immersion objective (Nikon Plan Apo VC 60 × A/1.20 WI) and illumination with 488 and 637 nm an NV laser unit. Images were collected at 30 fps with a A1-DUVB GaAsP detector unit with variable emission (400–720 nm) and NIS-Elements software, version 5.10 (Nikon). At least 5 experiments per condition were performed.

### Calculation of curvature induction

Classical molecular dynamics simulations of ANXA6 with 5 calcium ions associated with it on a lipid bilayer were performed using Gromacs 2020.x^37^ and the Charmm36 force field^38^. The initial coordinates for ANXA6 were obtained from the crystal structure of the phosphorylation-mimicking mutant T356D (PDBID: 1M9I). The mutation was reversed in the simulations. The crystal structure had 3 calcium ions bound to one annexin domain, and two to the other. CHARMM-GUI^39^ was used to construct a symmetric lipid bilayer composed of 680 lipid molecules with a POPC:POPS ratio of 4:1. The TIP3P water model was used to solvate the system and the system was neutralized with 150 mM KCl. We also ran a 1000 ns control simulation of a bilayer without ANXA6. The simulation run parameters were similar to those described in our previous investigations of annexins^34,40^. Three replicas for the system were simulated for 1000 ns each. Visual Molecular Dynamics (VMD) was used for visualization^41^.

### Model for rolling length

The model for rolling as described in detail in Boye et al*.*^6^ was used for estimating the variation in rolling length *L* as a function of: Spontaneous curvature *c*_*0*_ induced by annexin binding, adhesion energy *w*_*ad*_ between the patch membrane and the underlying membranes, the mean curvature elastic modulus *k*_*c*_ and the slope *b* of the roll spiral. Here we use the model to specifically examine how changes to *w*_*ad*_ and *c*_*0*_ may influence the rolling length and potentially explain observations of incomplete rolling. The value of *k*_*c*_ was kept fixed at *k*_*c*_ = 4.0 × 10^–20^ J and the value of *b* kept at *b* = 2.1 nm.

### Supported lipid bilayer preparation for AFM experiments

The supported lipid bilayer (SLB) preparation method has been described elsewhere^42^. Briefly, DOPC and DOPS were solubilized in methanol at a ratio of 1:1 with DiD-C18 probe added at 0.5% (Thermo, Invitrogen). Solubilized mixed lipids were dried by a nitrogen flow for 30 min. Further drying was achieved by storing lipids in a vacuum chamber overnight. Dried lipids were hydrated in 10 mM TRIS buffer (2-Amino-2-(hydroxymethyl)propane-1,3-diol), 140 mM NaCl, 2 mM Ca^2+^, pH = 7.4 at 55 °C for 2 h and then vortexed to form multilamellar vesicles. The resulting solution was tip sonicated for 10 min at 30 W in an ice/water bath to form small unilamellar vesicles (SUVs). SUVs were deposited on freshly cleaved mica secured in an AFM fluid cell, to form a supported lipid bilayer (SLB). After 15 min, excess lipids were rinsed away with buffer.

### Atomic force microscopy

Atomic force microscopy (AFM) was performed using a Nano Wizard 4 (JPK, Bruker) operated in alternating contact mode. Ultra-short cantilevers (USC-F0.3-k0.3, Nano World) with a nominal spring constant of 0.35 Nm^−1^ and a probe radius of 10 nm (according to manufacture specifications) were used. All scanning was performed using the smallest possible contact force to minimize potential sample deformation. For each experiment, annexins were added to SLBs in 10 mM TRIS buffer (pH 7.4) from a known concentrated solution such that the final bulk concentration in the fluid cell (made in-house) was 40 nM. An equilibration time of 10 min was allowed after addition of annexins prior to imaging. For each condition, 3 independent experiments were performed. Processing of AFM images was done with JPK Data Processing software, version 7.0.145 (JPK, Bruker).

## Supplementary Information

Supplementary Figures.

## Data Availability

The datasets used and analysed during the current study are available from the corresponding author on reasonable request.
