# Supplementary material for: Interplay of membrane crosslinking and curvature induction by annexins
Source: Sci Rep. 2022 Dec 29;12:22568. doi: 10.1038/s41598-022-26633-w (PMC9800579; doi:10.1038/s41598-022-26633-w)
Supplement: Supplementary file 1 — Supplementary Figures. [file 41598_2022_26633_MOESM1_ESM.pdf]

Supplemental Information for 'Interplay of membrane crosslinking by Annexins A1, A2, A6 with the curvature induction of Annexin A4: Implications for plasma membrane repair'

Anna Mularski[1], Stine Lauritzen Sønder[2], Anne Sofie Busk Heitmann[2], Mayank Prakash Pandey[1], Himanshu Khandelia[1], Jesper Nylandsted[2], and Adam Cohen Simonsen[1]*

[1]Department of Physics, Chemistry and Pharmacy, University of Southern Denmark, Campusvej 55, DK-5230 Odense M. Denmark.

[2]Membrane Integrity, Danish Cancer Society Research Center, Strandboulevarden 49, DK-2100 Copenhagen, Denmark.

* corresponding author: adam@sdu.dk

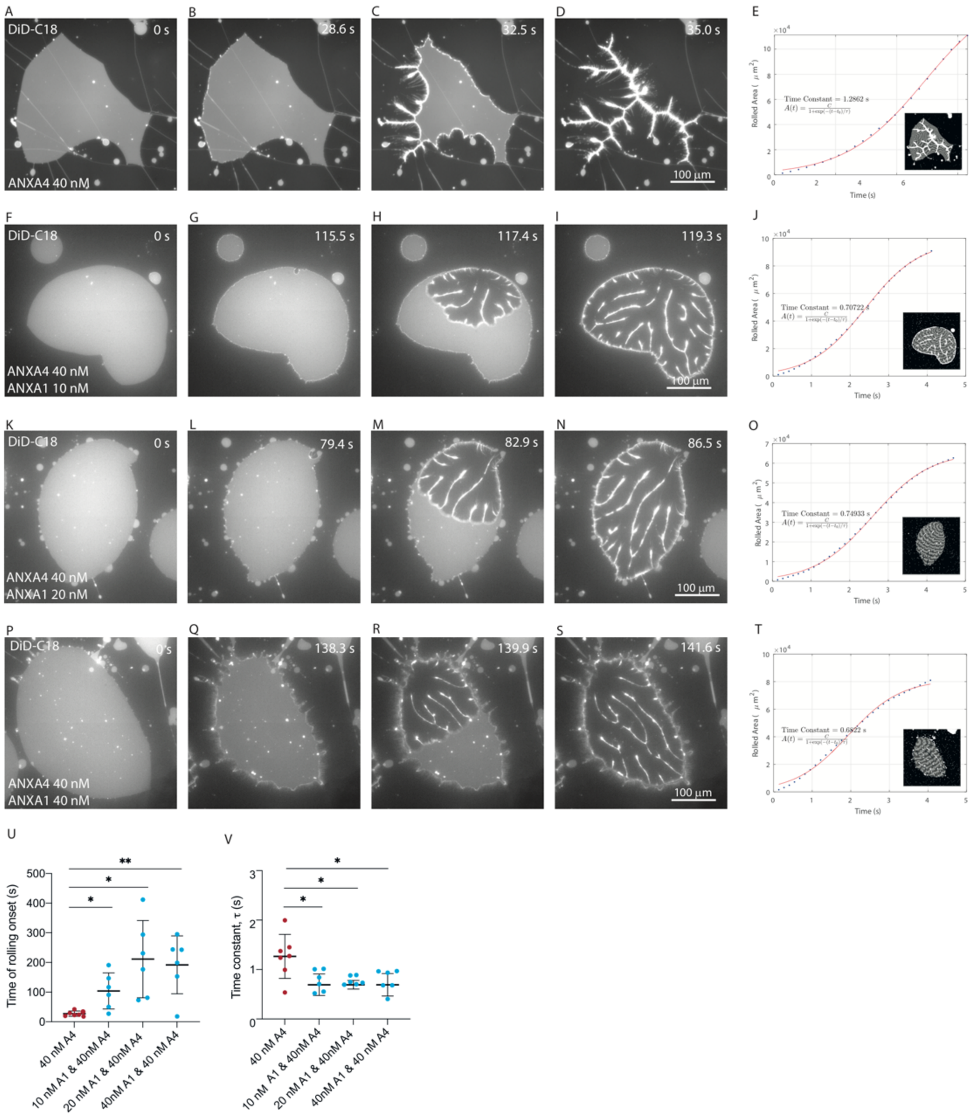

SI Figure 1: Response of non-vesicular membrane patches with open edges (DOPC/DOPS, 9:1 molar ratio) to ANXA4 and ANXA1 mixtures. 40 nM ANXA4 (A – D), 40 nM ANXA4 and 10 nM ANXA1 (F – I), 40 nM ANXA4 and 20 nM ANXA1 (K – N), 40 nM ANXA4 and 40 nM ANXA1 (P-S). In all cases (N > 6), a complete roll up of the membrane patch was observed. Quantification of the incremental area reduction between image frames is shown graphically (E, J & O respectively) where alternating dark and light grey bands show area reduction during rolling. Plotted as cumulative rolled area (blue circles, E, J O & T), the rolling time constant, tau, was derived from a fit to a logistic function (red line, E, J, O & T). Rolling time constants for 40 nM ANXA4 & 10, 20 & 40 nM ANXA1 (light blue circles, U) are significantly shorter (Welch's t test, * P < 0.05) than 40 nM ANXA4 (red circles, U). The time of rolling onset (light blue circles, V) is significantly longer for 40 nM ANXA4 and 10 nM ANXA1 (*), 40 nM ANXA4 and 20 nM ANXA1 (*) and 40 nM ANXA4 and 40 nM ANXA1 (**)
than for 40 nM ANXA4 (red circles, V).

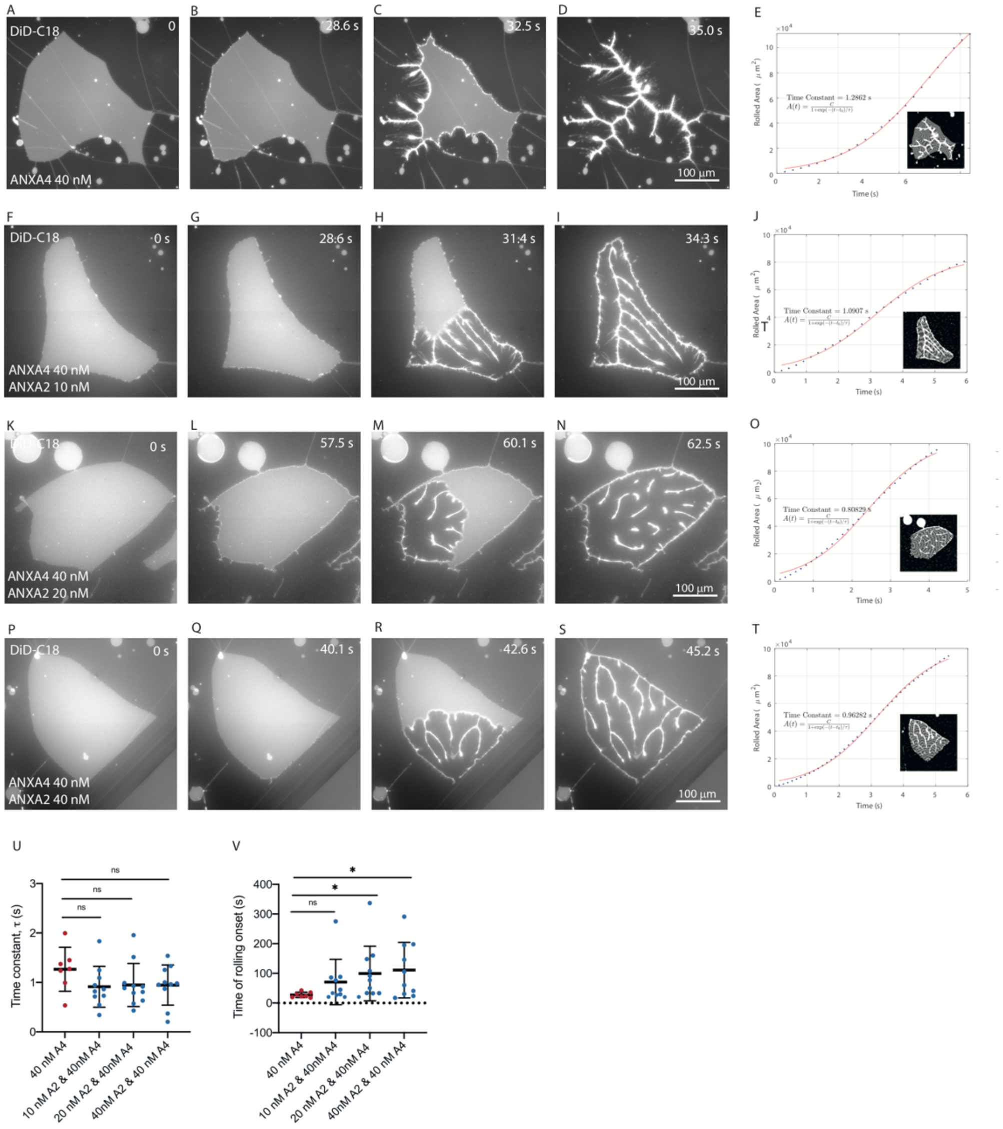

SI Figure 2: Response of non-vesicular membrane patches with open edges (DOPC/DOPS, 9:1 molar ratio) to ANXA4 and ANXA2 mixtures. 40 nM ANXA4 (A – D), 40 nM ANXA4 and 10 nM ANXA2 (F – I), 40 nM ANXA4 and 20 nM ANXA2 (K – N), 40 nM ANXA4 and 40 nM ANXA2 (P-S). In all cases (N > 7), a complete roll up of the membrane patch was observed. Quantification of the incremental area reduction between image frames is shown graphically (E, J & O respectively) where alternating dark and light grey bands show area reduction during rolling. Plotted as cumulative rolled area (blue circles, E, J O & T), the rolling time constant, tau, was derived from a fit to a logistic function (red line, E, J, O & T). Rolling time constants for 40 nM ANXA4 & 10, 20 & 40 nM ANXA1 (blue circles, U) were similar (ns) to 40 nM ANXA4 (red circles, U). The time of rolling onset (blue circles, V) is significantly longer for 40 nM ANXA4 and 20 nM ANXA2 (Welch's t test, *P < 0.05) than 40 nM ANXA4 and 40 nM ANXA2 (*) than for 40 nM ANXA4 (red circles, V) but not 40 nM ANXA4 and 10 nM ANXA2 (ns).

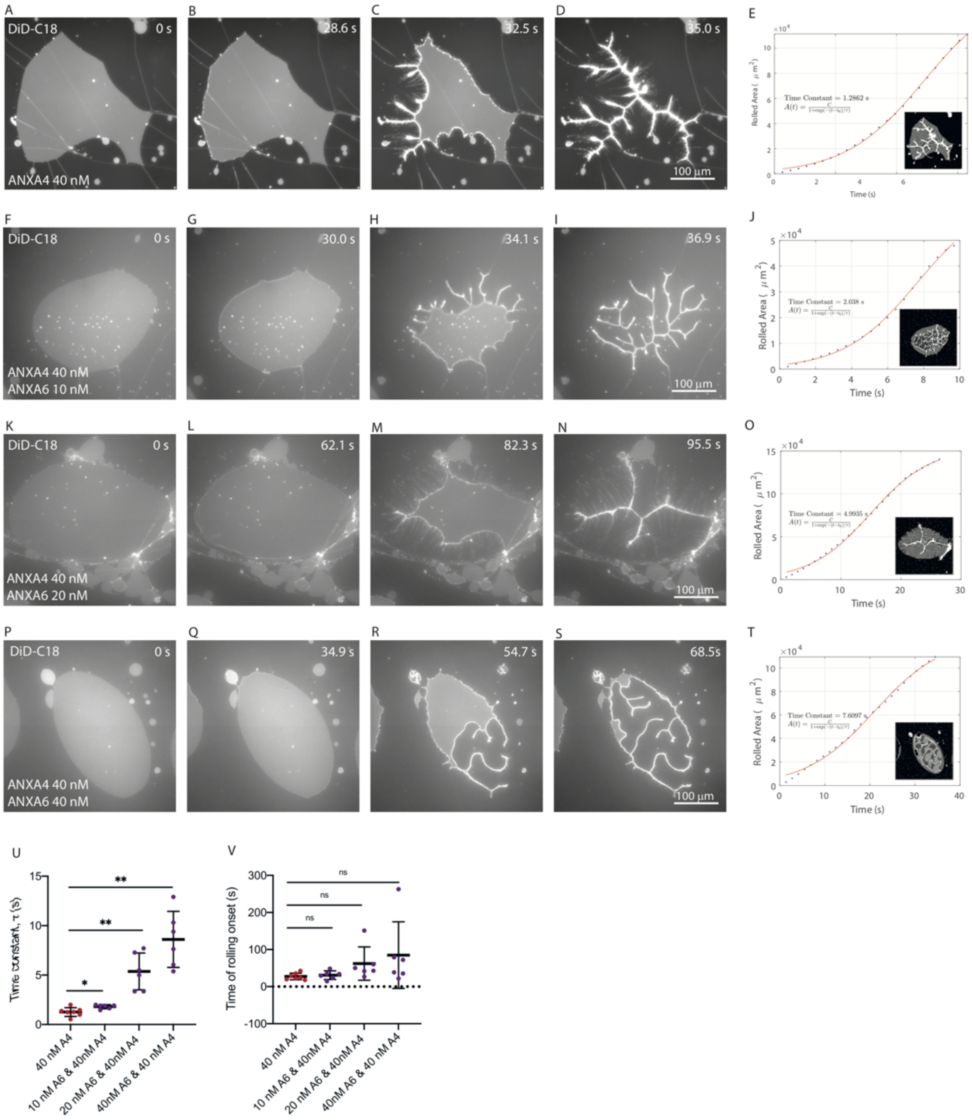

SI Figure 3: Response of non-vesicular membrane patches with open edges (DOPC/DOPS, 9:1 molar ratio) to ANXA4 and ANXA6 mixtures. 40 nM ANXA4 (A – D), 40 nM ANXA4 and 10 nM ANXA6 (F – I), 40 nM ANXA4 and 20 nM ANXA6 (K – N), 40 nM ANXA4 and 40 nM ANXA6 (P-S). In all cases (N > 6) patches underwent membrane rolling. Quantification of the incremental area reduction between image frames is shown graphically (E, J & O respectively) where alternating dark and light grey bands show area reduction during rolling. Plotted as cumulative rolled area (blue circles, E, J O & T), the rolling time constant, tau, was derived from a fit to a logistic function (red line, E, J, O & T). Rolling time constants for 40 nM ANXA4 & 10, 20 & 40 nM ANXA6 (purple circles, U) longer than 40 nM ANXA4 (red circles, U) (10 nM ANXA6, Welch's t test, *P < 0.05; 20 nM ANXA6, **; 40 nM ANXA6, **). The time of rolling onset for 40 nM ANXA4 & 10, 20 & 40 nM ANXA6 (purple circles, V) is similar (ns) to 40 nM ANXA4 (red circles, V).

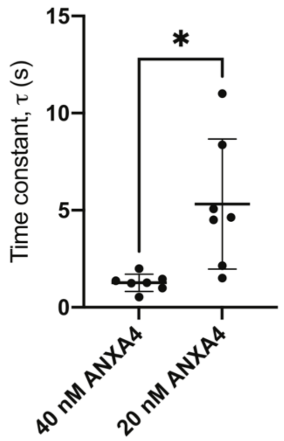

SI Figure 4: Rolling time constants for membrane patch experiments performed with 40 nM and 20 nM ANXA4. Time contstants in the presence of 40 nM ANXA4 are significantly shorter than those in the presence of 20 nM ANXA4 (Welch's t test, *P < 0.05).

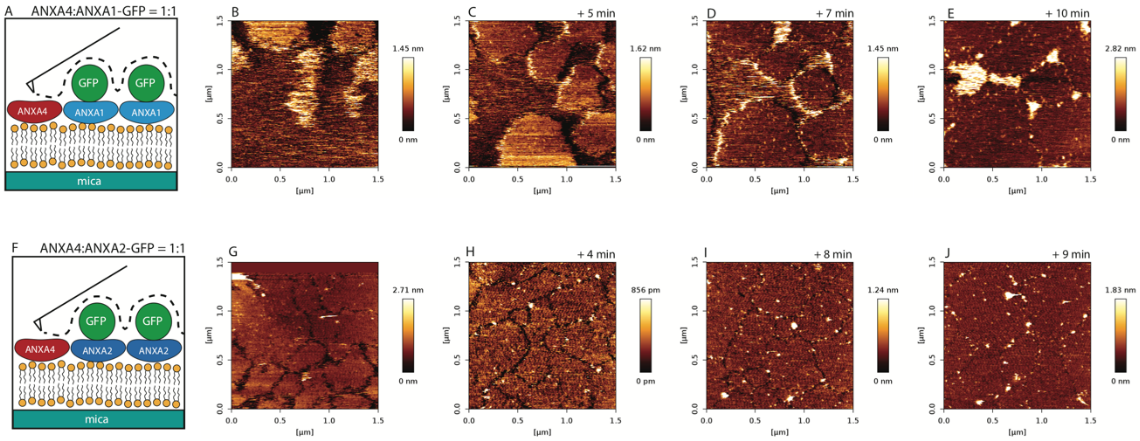

SI Figure 5: Crystallization of ANXA4 in the presence of ANXA1 and ANXA2. Schematic representation of AFM imaging of proteins structures that result when 1:1 annexin mixtures (ANXA4 and ANXA1-GFP (A), ANXA4 and ANXA2-GFP (F) are added to DOPC/DOPS (1:1 molar ratio) supported lipid bilayers. The presence of the GFP label on provides a height differential between the two proteins. A time course of 1.5 um images for ANXA1-GFP and ANXA4 (B-E) and ANXA2-GFP and ANXA4 (G-J) indicates ANXA4 crystallization precedes binding of GFP labelled crosslinker protein.

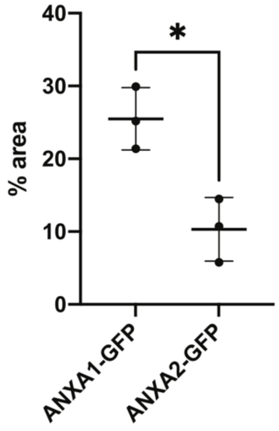

SI Figure 6: Proportion of membrane area occupied by ANXA1-GFP and ANXA2-GFP in the presence of ANXA4. ANXA1-GFP binds significantly more effectively to flat membranes in the presence of ANXA4 than ANXA2-GFP (Welch's t test, *$P < 0.05$).
